# Atypical Presentation of IgA Nephropathy Mimicking Acute Pyelonephritis

**DOI:** 10.1155/2018/9231989

**Published:** 2018-06-13

**Authors:** Stamatis Karakonstantis, Despoina Galani, Dafni Korela, Sofia Pitsigavdaki, Ifigeneia Kassotaki, Despoina Arna, Dimitrios Xydakis

**Affiliations:** ^1^2nd Department of Internal Medicine, General Hospital of Heraklion “Venizeleio-Pananeio”, Heraklion, Greece; ^2^Nephrology Department, General Hospital of Heraklion “Venizeleio-Pananeio”, Heraklion, Greece

## Abstract

**Background:**

IgA glomerulonephritis may present with hematuria, flank pain, and fever. This clinical presentation may be easily confused with acute pyelonephritis.

**Case Report:**

We present the case of a 25-year-old female with a typical clinical presentation for acute pyelonephritis (high fever, left flank pain, left costovertebral angle tenderness, hematuria, elevated inflammatory markers, and a hypoenhancing region in the left kidney on contrast-enhanced computed tomography). However, urine and blood cultures were both negative, the serum creatinine was elevated, and the urinalysis revealed significant proteinuria and dysmorphic red blood cells. A kidney biopsy confirmed a diagnosis of IgA nephropathy. She was treated with a combination of lisinopril and methylprednisolone, with good response.

**Conclusion:**

Gross hematuria, especially in the absence of pyuria or bacteriuria, should raise the suspicion for underlying IgA nephropathy, even if the rest of the clinical presentation is typical for a urinary tract infection. The presence of significant proteinuria, red blood cell casts, and dysmorphic red blood cells are useful clues suggesting glomerular disease.

## 1. Introduction

IgA glomerulonephritis may present with gross hematuria and flank pain [1, 2]. Fever may also be present, possibly because most episodes of IgA nephropathy-associated gross hematuria coincide with infections, especially upper respiratory tract infections [1, 2]. The combination of fever, flank pain, and hematuria may be easily confused with acute pyelonephritis. Here, we present an unusual case of a young female with a clinical presentation and imaging typical for acute pyelonephritis that however was diagnosed with IgA nephropathy.

## 2. Case report

A 25-year-old female with no prior medical history presented to the emergency department due to high fever (up to 39°C) since 3 days. She complained of left flank pain and gross hematuria. On physical examination, left costovertebral angle tenderness was noted. The urinalysis confirmed the hematuria (2055 red blood cells per high-power field). The urinary dipstick was negative for leukocyte esterase and for nitrites, but significantly positive for albuminuria (2+). On microscopic examination of the urine, pyuria was minimal (6 white blood cells per high-power field) and no bacteriuria was noted. She denied having taken antibiotics before presentation. Urine and blood cultures were obtained. The laboratory tests revealed a significantly elevated C-reactive protein (CRP = 28 mg/dl), a high erythrocyte sedimentation rate (107 mm/h), and an elevated creatinine (1.21 mg/dl) with normal blood urea nitrogen (16 mg/dl).

**Figure 1:**
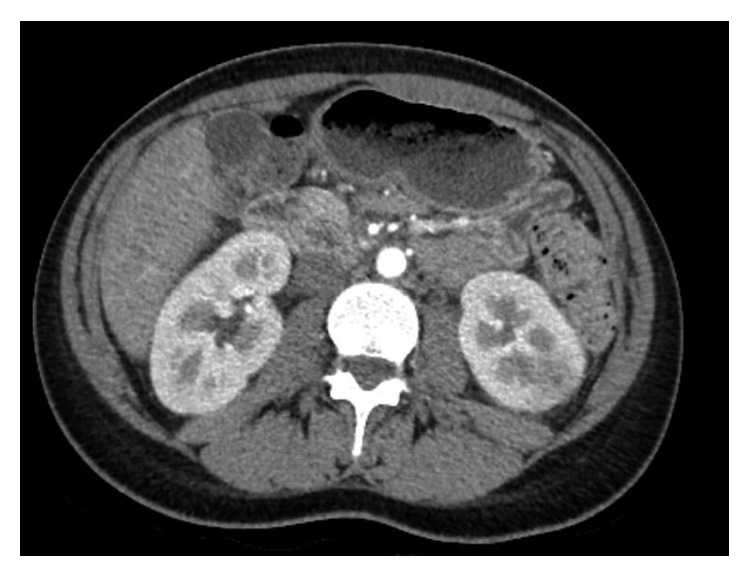
Contrast-enhanced computed tomography demonstrating a hypoenhancing region in the left kidney.

She was admitted to the internal medicine ward with a preliminary diagnosis of acute pyelonephritis, and she was started on intravenous ceftriaxone. A contrast-enhanced computed tomography the next day revealed a hypoenhancing region in the upper pole of the left kidney, suggestive of pyelonephritis (Figure 1). However, considering the significant hematuria in the absence of pyuria and bacteriuria, and the persistently elevated creatinine (1.55 mg/dl on day 3), a nephrologist was consulted. Microscopic evaluation of the urinary sediment revealed dysmorphic red blood cells suggesting glomerular disease (2 red blood cell casts and 60–80 red blood cells per high-power field with >10% of G1 cells and >80% dysmorphic erythrocytes). The spot urine protein to creatinine ratio obtained on the 5th day of hospital stay was also elevated (929 mg/g). Furthermore, both urine and blood cultures came back negative, and no fever was recorded during the hospital stay. Ceftriaxone was discontinued after 7 days of treatment.

A biopsy of the left kidney was performed at the 6th day of hospital stay and confirmed a diagnosis of IgA nephropathy (immunofluorescence was strongly positive for mesangial IgA deposition, complement component C3, and lambda light chains and moderately positive for IgM). The patient denied any recent respiratory tract infection symptoms. Other lab tests sent during her hospital stay were normal (lactate dehydrogenase, anti-neutrophil cytoplasmic antibodies, antinuclear antibodies, anticardiolipin IgG and IgM, anti-beta-2 glycoprotein I IgG and IgM, protein electrophoresis, serum complement C3c and C4 level, rheumatoid factor, and transesophageal echocardiography). She was started on lisinopril 5 mg daily. Furthermore, considering the significant proteinuria, the elevated creatinine and the negative prognostic features of the biopsy, (M1E1S1T0C1 according to the Oxford classification [3]) she was started on glucocorticoids (three-day pulse of methylprednisolone 1g in months 1, 3, and 5 in addition to oral prednisolone 0.5 mg/kg every other day for 6 months) [4]. At follow-up at 2 months, both creatine (0.86 mg/dl) and the spot urine protein to creatinine (114 mg/g) were normal. CRP at follow-up was 0.4 mg/dl.

## 3. Discussion

Despite a clinical presentation typical for pyelonephritis (high fever, flank pain, and costovertebral angle tenderness) and compatible imaging (computed tomography) and laboratory tests (high CRP), our patient was diagnosed with IgA nephropathy, confirmed by kidney biopsy. The lack of pyuria, the negative dipstick for nitrites and leukocyte esterase, and the fact that both urine and blood cultures were negative, despite both being obtained before initiation of antimicrobial therapy, put in doubt the diagnosis of pyelonephritis. Nevertheless, the negative predictive value of urinalysis may be lower [5] than previously reported [6, 7]. Furthermore, the possibility of culture-negative pyelonephritis cannot be excluded [8, 9].

Possible reasons for culture-negative pyelonephritis include antibiotic pretreatment, difficult to culture atypical microorganisms such as *Ureaplasma urealyricum* or *Mycoplasma hominis*, bacteriuria below the defined cutoffs of clinically significant bacteriuria, or infection confined to the renal parenchyma as result of hematogenous seeding [8]. The cutoff for reporting bacteriuria in our microbiology laboratory is 10^3^ colony forming units/ml; therefore, bacteriuria below this level would have not been reported. The possibility of infection by *Ureaplasma urealyricum* or *Mycoplasma hominis*, although unlikely, cannot be ruled out. Nevertheless, our patient improved without receiving antibiotic treatment to cover these pathogens. Isolated parenchymal disease also cannot be excluded, despite the negative blood cultures, considering that the yield of blood cultures is generally low [10].

The presence of significant proteinuria was another important clue for reaching the correct diagnosis in our patient. Transient low-level proteinuria can also occur due to fever or urinary tract infection [11]. However, the level of proteinuria in our patient was high and persistent despite the resolution of fever (929 mg/g on the 5th day of hospital stay).

The very high CRP in our case is an unexpected finding for IgA nephropathy in the absence of infection, since such high elevations have not been described in prior studies [12–14]. It is unclear however if in these studies any measurements were obtained during an acute episode of gross hematuria, like in our case. An infection could not be proven in our case. The patient denied any respiratory symptoms, and a urinary tract infection was not confirmed given the negative urinalysis and cultures. Whether these findings (high CRP and high-grade fever) in our case could be solely attributed to an exacerbation of IgA nephropathy or if a concurrent unidentified infection or culture-negative pyelonephritis was also present is unclear.

The imaging on computed tomography in our patient is also unusual for IgA nephropathy. However, focal hypoenhancing areas on computed tomography in the setting of IgA nephropathy have been described by other authors too [15]. Such lesions could be attributed to focal acute tubular necrosis as a result of obstruction and tubular damage caused by the hematuria [15]. Whether this finding is causal or coincidental requires further studying. The differential diagnosis of the CT in our patient includes renal infarction, which may also present with the combination of gross hematuria, flank pain, and fever [16]. Typically, renal infarctions are associated with an elevated lactate dehydrogenase and wedge-shaped hypoenhancing lesions with the cortical rim sign on CT [16]. None of these findings were present in our case, and our patient did not have any risk factors for thromboembolic renal infarction.

Of note is that our patient reported a similar prior episode in the past (fever accompanied by hematuria) at which time she had been treated with antibiotics, and no further work-up was performed. Although it is unclear if that episode represented the first manifestation of IgA nephropathy, confirming the diagnosis of IgA nephropathy at that time could have prevented long-term kidney damage.

In conclusion, even in cases with clinical presentation typical for pyelonephritis, gross hematuria should raise the suspicion for underlying IgA nephropathy, especially in the absence of pyuria or bacteriuria and when there is acute worsening of renal function. In such cases, it is reasonable to perform a microscopic examination of the urinary sediment looking for signs of glomerular disease (especially red blood cell casts and dysmorphic red blood cells). Misdiagnosing IgA nephropathy-associated episodes of hematuria as urinary tract infections may lead to long-term irreversible kidney damage.
